# ^89^Zr, a Radiometal Nuclide with High Potential for Molecular Imaging with PET: Chemistry, Applications and Remaining Challenges

**DOI:** 10.3390/molecules18066469

**Published:** 2013-06-03

**Authors:** Gabriel Fischer, Uwe Seibold, Ralf Schirrmacher, Björn Wängler, Carmen Wängler

**Affiliations:** 1 Biomedical Chemistry, Department of Clinical Radiology and Nuclear Medicine, Medical Faculty Mannheim of Heidelberg University, Mannheim 68167, Germany; E-Mails: Gabriel.Fischer@medma.uni-heidelberg.de (G.F.); Uwe.Seibold@medma.uni-heidelberg.de (W.S.); 2 University Hospital Munich, Department of Nuclear Medicine, Ludwig Maximilians-University, Munich 81377, Germany; 3 Molecular Imaging and Radiochemistry, Department of Clinical Radiology and Nuclear Medicine, Medical Faculty Mannheim of Heidelberg University, Mannheim 68167, Germany; E-Mail: Bjoern.Waengler@medma.uni-heidelberg.de; 4 McConnell Brain Imaging Centre, Montreal Neurological Institute, McGill University, Montreal, QC H3A 2B4, Canada; E-Mail: Ralf.Schirrmacher@mcgill.ca

**Keywords:** biomolecules, Cerenkov luminescence imaging, desferrioxamine B, dosimetry, positron emission tomography (PET), ^89^Zr

## Abstract

Molecular imaging—and especially Positron Emission Tomography (PET)—is of increasing importance for the diagnosis of various diseases and thus is experiencing increasing dissemination. Consequently, there is a growing demand for appropriate PET tracers which allow for a specific accumulation in the target structure as well as its visualization and exhibit decay characteristics matching their *in vivo* pharmacokinetics. To meet this demand, the development of new targeting vectors as well as the use of uncommon radionuclides becomes increasingly important. Uncommon nuclides in this regard enable the utilization of various selectively accumulating bioactive molecules such as peptides, antibodies, their fragments, other proteins and artificial structures for PET imaging in personalized medicine. Among these radionuclides, ^89^Zr (t_1/2_ = 3.27 days and mean E_β+_ = 0.389 MeV) has attracted increasing attention within the last years due to its favorably long half-life, which enables imaging at late time-points, being especially favorable in case of slowly-accumulating targeting vectors. This review outlines the recent developments in the field of ^89^Zr-labeled bioactive molecules, their potential and application in PET imaging and beyond, as well as remaining challenges.

## 1. Introduction

The development of new radiotracers for application in personalized medicine with PET has experienced enormous progress over the last decade, which is reflected in the large and still growing number of valuable compounds used for imaging of different diseases. A tendency that can be observed in this PET tracer development is the utilization of labeled targeting vectors that accumulate with high specificity at the target site, allowing for a highly sensitive target visualization. Many of these vectors are relatively large structures exhibiting high molecular weights as well as slow pharmacokinetics, necessitating the use of radionuclides with long half-lives in order to fully exploit the potential of the used targeting vectors for diagnostic imaging.

Interest in ^89^Zr has increased over the last several years as it exhibits a long half-life, making it ideally suited for imaging studies with slowly-accumulating bioactive molecules, allowing for imaging of biological processes at late time-points after tracer application. ^89^Zr—about which excellent other reviews are available [1,2,3,4]—produces positrons with a probability of 22.3% [5,6] and a mean energy of 0.389 MeV, which is between the positron energies of ^18^F (mean β^+^ energy of 0.250 MeV) and ^68^Ga (mean β^+^ energy of 0.836 MeV) [7], and thus allows for high resolution PET images. ^89^Zr is produced via cyclotron using ^89^Y (which has a natural abundance of 100%) as target material in the ^89^Y(p,n)^89^Zr nuclear reaction [8,9,10,11,12,13] and can be obtained in high isolated yields of 94–99.5% and very high radionuclidic purity of 99.99% [5,8].

Although ^18^F exhibits very favorable physical decay characteristics of 96.7% positron emission probability and a low positron energy, it cannot be used for *in vivo* imaging of biomolecules with slow pharmacokinetics due to its rather short half-life of 109 min. Thus, the use of ^18^F, as well as that of ^68^Ga (t_1/2_ = 68 min) and ^11^C (t_1/2_ = 20 min) which are the common nuclides in routine PET imaging applications, is restricted to imaging of the biodistribution of smaller radiolabeled bioactive compounds such as peptides and small molecules. As a result, ^64^Cu has attracted attention as it exhibits a longer half-life of 12.7 h together with a main positron energy of 0.653 MeV and low spatial resolution loss of 0.7 mm, allowing for an extended imaging of biological processes. However, ^89^Zr is even more interesting for the radiolabeling of slowly-accumulating radiopharmaceuticals as it exhibits a longer half-life of 3.27 days, being particularly suited for *in vivo* imaging of antibodies, nanoparticles and other large bioactive molecules [14,15]. However, this results in higher absorbed organ doses than in case of ^18^F-labeled tracers such as [^18^F]FDG [16,17]. ^124^I could also serve as a possible alternative for long-term imaging, exhibiting a half-life of 4.18 days, but in comparison to ^89^Zr it exhibits higher main positron energies of 1.54 and 2.14 MeV resulting in a relatively high intrinsic spatial resolution loss of 2.3 mm, whereas ^89^Zr with an intrinsic spatial resolution loss of 1.0 mm gives much better imaging results [18]. Furthermore, ^124^I is a so-called non-pure positron-emitter, producing in significant amount high-energy photons of different energy (603 keV (63.0%), 1691 keV (10.9%) and 723 keV (10.4%) [7]) which can result in random and scatter non-true coincidences and thus background noise, whereas ^89^Zr produces mainly one additional γ-line at 909 keV (909 keV (99.9%), 1657 (0.1%), 1713 keV (0.77%) and 1.744 keV (0.13%) [19]). Thus, the application of ^124^I necessitates the use of appropriate image reconstruction techniques in order to achieve a reasonable image quality [7,20].

Although ^89^Zr is mainly used for *in vivo* PET imaging, its use is not restricted to PET alone, as it can also be used for *in vivo* Cerenkov luminescence imaging (CLI) [21,22]. This imaging modality is based on luminescence that can be observed when a particle travels faster than light in the examined medium, is fully quantifiable and can be correlated to the respective PET signal [22]. Thus, Cerenkov luminescence imaging was shown to be applicable in image-guided surgery using ^89^Zr-labeled antibodies [23]. However, the main application of ^89^Zr is–and probably will remain–*in vivo* PET imaging of biological processes. Among ^89^Zr-labeled bioactive molecules used for PET imaging, mainly antibodies were labeled with ^89^Zr so far [24,25,26,27,28,29] although its use is not limited to these biomolecules. Beyond antibodies, ^89^Zr-labeling was also shown to be applicable in long-term PET studies of peptides and peptide multimers [30], nanoparticles [31], microspheres [32], targeted nanotubes [33], liposomes [34] and proteins [35,36].

In the following paragraphs, the radiolabeling with ^89^Zr, the synthesis strategies to introduce desferrioxamine B (DFO) into bioactive molecules, the stability of the ^89^Zr-DFO complex and its chemical conjugation as well as an overview of ^89^Zr-labeled biomolecules and their applications will be presented and critically discussed.

## 2. Radiolabeling of Bioactive Molecules with ^89^Zr

Although direct labeling approaches without a chelator have been described for ^89^Zr-introduction into liposomes [34] and serum proteins [37], reasonable labeling approaches with ^89^Zr have to be carried out using an appropriate chelator system in order to prevent the liberation of the radionuclide from the targeting vector. Liberated ^89^Zr^4+^ can bind to plasma proteins [38] and accumulates in mineral bone over time depositing significant doses there [39,40] which has to be avoided.

Diethylenetriaminepentaacetic acid (DTPA) was shown to not stably bind ^89^Zr [41], even though it exhibits a higher complex stability than the corresponding ethylenediaminetetraacetic acid (EDTA) complex [42]. Desferrioxamine B (a chelating agent binding various metal-ions [43,44]) was introduced for ^89^Zr complexation in 1992 and found to exhibit superior properties compared to DTPA [41]. DFO is still the commonly used chelator for ^89^Zr-complexation and is conjugated to the biomolecule before the radiolabeling reaction takes place.

Desferrioxamine B is commercially available in different chemical forms as DFO salt (e.g., from Sigma Aldrich, Schnelldorf, Germany) or in reactive form as the isothiocyanate derivative (Macrocyclics, Dallas, TX, USA), the maleimide derivative (Macrocyclics, Dallas, TX, USA) and the tetrafluorophenolic active ester chelated with Fe^3+^ (ABX, Radeberg, Germany). ^89^Zr is also commercially available on a regular basis from IBA Molecular (Richmond, VA, USA) and BV Cyclotron VU (Amsterdam, The Netherlands).

**Scheme 1:**
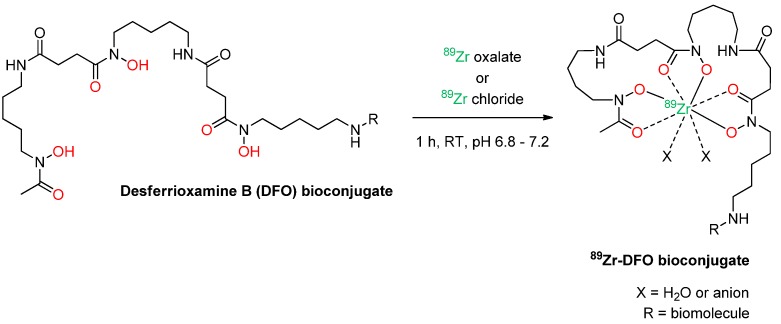
Schematic depiction of the radiolabeling reaction of a DFO-derivatized biomolecule with ^89^Zr (in red: hydroxamate coordination sphere of DFO).

In the desferrioxamine B complex, the oxophile Zr^4+^ ion is supposed to be stabilized by the three hydroxamates and two additional anions or water molecules resulting in an expansion of the first coordination sphere, geometry relaxation and an octadentate complex structure [45] (Scheme 1). However, instead of a crystal structure, only DFT calculations of the ^89^Zr-DFO-complex are available so far [46].

A typical procedure for ^89^Zr-labeling consists of the following steps: (*i*) dilution of the ^89^Zr oxalic acid solution with saline or oxalic acid solution and adjustment of the pH of the solution to the optimal pH-range of 6.8 to 7.2 [47] by addition of Na_2_CO_3_ and/or HEPES buffer; (*ii*) addition of the DFO-derivatized bioactive compound and reaction for 30-60 min at ambient temperature; and (*iii*) purification of the radiolabeled product via HPLC, size exclusion chromatography or ultrafiltration. The radiolabeled products can be obtained in variable yields between 35% [48] and 98% [24], but in the vast majority of cases, radiochemical yields exceeding 75% are observed [8,26,47,49]. When presented, the radiochemical purities (RCPs) of the products were consistently reported to be over 95% after purification. However, upon storage, the reported RCPs strongly vary from no decrease in RCP after several days of storage in saline or buffered solution [8,26,46,50,51,52] to a significant 5% to 20% decrease in RCP after 48h or 168h [8,24,53]. DTPA was shown to significantly challenge the ^89^Zr-DFO-complex resulting in a 25–45% decrease in product RCPs after 7 days [5,50]. Other effects can also decrease the RCP of a ^89^Zr-preparation upon storage. Factors such as storage medium, pH as well as the applied temperature were found to significantly influence the RCP of the ^89^Zr-radiotracer over time and can result in a reduction of RCP of up to 48% after 144 h [47].

## 3. Introduction of Desferrioxamine B into Bioactive Molecules

Although the chelator chemistry available for ^89^Zr complexation is limited to desferrioxamine B alone, the introduction of this chelator into bioactive molecules can be carried out using different approaches. The most commonly used ones are based on acid-amide coupling and thiourea-formation, although also other methodologies such as click chemistry reactions can be used (see below). The different DFO conjugation approaches do not only result in different conjugation and radiolabeling efficiencies, but also in partially differing biodistribution properties of the radiolabeled compounds. 

### 3.1. Introduction of DFO via Thiol Conjugation

**Scheme 2:**
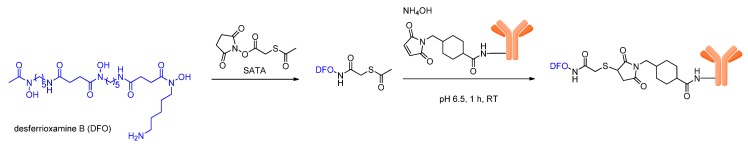
Schematic depiction of the conjugation reaction of thiol-derivatized DFO to maleimide-modified biomolecules.

One of the first described possibilities to introduce DFO into bioactive molecules is via the addition of thiols to maleimides. For this purpose, DFO can be reacted with *N*-succinimidyl-*S*-acetylthioacetate (SATA), resulting in an *S*-acetyl-protected thiol derivatization of the chelator (Scheme 2). In parallel, the biomolecule is reacted with 4-(*N*-maleimidomethyl) cyclohexane carboxylic acid *N*-hydroxysuccinimide ester (SMCC) to introduce maleimide moieties. Subsequently, both derivatized compounds can, in the presence of hydroxylamine, be reacted at physiologic pH to yield the DFO-biomolecule-conjugate (Scheme 2). Different antibody-chelator-conjugates were prepared using this method, labeled with ^89^Zr and evaluated in tumor-bearing mice, all showing a bone accumulation of approximately 2–5% at 72 h *p.i.* which can be attributed to the liberation of ^89^Zr [54]. However, the ^89^Zr-immunoconjugates also accumulated significantly in the target tumor tissues and even showed a higher tumor accumulation than the reference antibodies labeled with ^123^I and ^99m^Tc after 72 h. Unfortunately, the ^89^Zr-labeled immunoglobulins also showed a higher background accumulation in other tissues, impairing the promising results. 

**Scheme 3:**
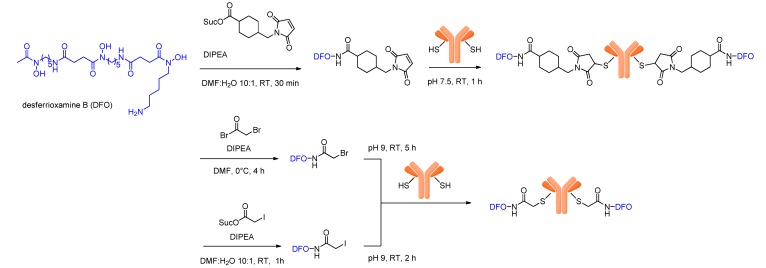
Schematic depiction of a site-specific DFO-conjugation by reaction of maleimide-, bromoacetyl- and iodoacetyl-derivatized DFO with biomolecules exhibiting unpaired cysteines.

Another approach utilizes antibodies bearing free thiols which can be reacted with thiol-reactive DFO derivatives. Different thiol-reactive DFO derivatives such as bromoacetyl-DFO, iodoacetyl-DFO and maleimidocyclohexyl-DFO were synthesized and could be conjugated to thiol-bearing trastuzumab which was recombinantly modified with unpaired cysteines that allowed for a site-specific conjugation of the thiol-reactive chelators (Scheme 3) [55].

The obtained ^89^Zr-labeled conjugates were evaluated *in vitro* and *in vivo* in a HER2-positive breast cancer mouse model and compared to non-site-specifically derivatized and ^89^Zr-labeled trastuzumab with regard to biodistribution and tumor visualization. Interestingly, the site-specific conjugation of the DFO chelator did not result in improved biodistribution properties compared to a non-site-specific modification using either the active ester or isothiocyanate approach (see section 3.3) for antibody derivatization as comparable *in vivo* properties were observed for all ^89^Zr-labeled immunoconjugates at different time points *p.i.*. This is astonishing as highly preserved antigen binding affinities of 0.87 ± 0.15 to 1.22 ± 0.22 nM could be found for the site-specifically modified antibodies (0.91 ± 0.15 nM *K_D_* for unmodified trastuzumab) which cannot be assumed in case of the non-site-specifically derivatized mAbs, although the respective data were not presented. 

Comparing the different thiol-reactive DFO derivatives in terms of reactivity, the conjugation efficiency varied. The best results were obtained for the maleimide-modified DFO whereas bromoacetyl- and iodoacetyl-DFO showed lower conjugation efficiencies and lower radiochemical purities after radiolabeling. In terms of *in vivo* stability and ^89^Zr-release, the different ^89^Zr-labeled antibodies gave similar results as reflected in similar bone accumulations that increased over time to 7–10% after 4 days *p.i.* [55].

### 3.2. Introduction of DFO via Click Chemistry

**Scheme 4:**
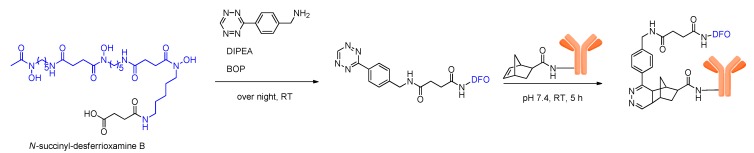
Schematic depiction of biomolecule-DFO-conjugation via Diels-Alder cycloaddition.

Another method to introduce DFO into antibodies was described recently, using a Diels-Alder click chemistry reaction between norbornenes and tetrazines on the example of trastuzumab [56]. Using this approach, the antibody and DFO were first reacted with a norbornene and a tetrazine moiety, respectively, before the conjugation of DFO to the antibody was carried out. This Diels-Alder conjugation reaction was performed under mild conditions at ambient temperature, but required relatively long reaction times of 5 h (Scheme 4).

The DFO-modified antibody was subsequently labeled with ^89^Zr and evaluated *in vitro* and *in vivo*. Besides the expected very favorable accumulation in the HER2-positive tumor that could be observed, which is mediated by the highly specific antibody, also a high *in vivo* bone accumulation of 13.2% ± 2.4% could be detected as early as 24 h *p.i.*. This is especially astonishing as the *in vitro* stability tests of the same labeled antibody in serum revealed a liberation of only ~2% of ^89^Zr from the complex after 7 days of incubation. As other ^89^Zr-labeled antibodies do not show such an extensive liberation of ^89^Zr from the complex within such a short period, this seems to be a result of a susceptibility of the conjugating linker which might also destabilize the ^89^Zr-DFO-complex making it sensitive to detachment and metabolization.

### 3.3. Introduction of DFO via Acid-Amide or Thiourea Formation

The direct conjugation of functionalized DFO to side chain amino groups of lysine is still the main approach for DFO introduction. Here, two different routes can be pursued: conjugation via an isothiocyanate or acid-amide coupling using an active ester of the chelator. 

**Scheme 5:**
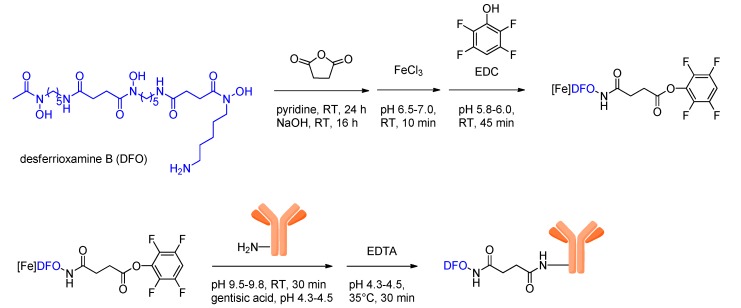
Schematic depiction of biomolecule-DFO-conjugation via reaction of amino functionalities of the biomolecule with a DFO-active ester.

The active ester strategy, first described by Verel *et al.* [8], is rather complex and consists of six steps: (*i*) succinylation of DFO; (*ii*) complexation of Fe^3+^ to prevent side-reactions involving the hydroxamate groups; (*iii*) formation of the tetrafluorophenolic active ester; (*iv*) conjugation of the DFO-active ester to the antibody; (*v*) removal of Fe^3+^ from the DFO complex by transchelation with EDTA (ethylenediamine tetraacetic acid) and *vi*) radiolabeling with ^89^Zr (Scheme 5).

Despite the rather complex synthesis strategy, this approach has the advantage of resulting in ^89^Zr-labeled antibodies exhibiting favorable *in vitro* and *in vivo* properties as was shown by a study directly comparing antibodies using the active ester and the thiol-maleimide approach described before (Scheme 2) [8]. The *in vitro* experiments for example revealed strongly differing stabilities in human serum for both radiopharmaceuticals. The antibody conjugate prepared by the acid-amide coupling showed no degradation within 24 h in human serum, whereas in the case of the thiol-maleimide conjugate, the ^89^Zr-DFO-complex was partially transferred from the antibody to serum albumin although not resulting in the liberation of the radiometal ion. It was suggested that this observed conjugation instability in case of the thiol-maleimide conjugation product could be due to a Zr-catalyzed opening of the succinimide ring. 

This observed complex-transfer from the antibody to albumin of course also resulted in differing *in vivo* imaging results for both antibodies. The antibody modified with DFO by the active ester strategy exhibited a longer blood pool circulation and also much higher tumor-to-background ratios than the thiol-maleimide-conjugate that showed a faster excretion and thus could not achieve the high tumor accumulation that was observed in case of the more stable acid-amide antibody-DFO conjugate. This study impressively shows that for obtaining a stable ^89^Zr-labeling resulting in radiopharmaceuticals with reasonable *in vivo* properties, the conjugation chemistry is a crucial factor. In this study–comparable to the results found by others–a significant bone accumulation resulting from ^89^Zr liberation could be observed that was similar for both antibodies [8]. 

Thus, the active ester strategy to introduce DFO into biomolecules is rather complex, but produces ^89^Zr-labeled compounds with favorable *in vitro* and *in vivo* properties and therefore is still one of the most often used methodologies for protein ^89^Zr-labeling [24,26,57,58,59]. Moreover, as the active ester complexed with Fe^3+^ (which can be directly applied for biomolecule modification) became commercially available recently, this approach for DFO-conjugation significantly gained simplicity, making it even more attractive for DFO-conjugation.

**Scheme 6:**
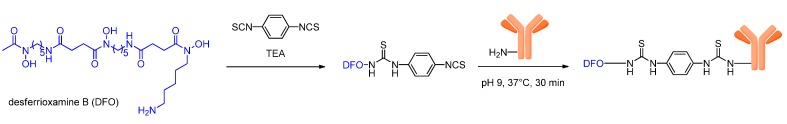
Schematic depiction of biomolecule-DFO-conjugation via reaction of lysine amino functionalities of the biomolecule with a DFO-isothiocyanate.

A methodology to efficiently introduce DFO into biomolecules was described recently and is based on a *p*-isothiocyanatobenzyl-derivative of DFO that can be directly conjugated at mildly basic pH within a relatively short reaction time of only ½ to 1 h (Scheme 6) and subsequently reacted with ^89^Zr to give the radiolabeled biomolecule [47].

The antibodies cetuximab and cU36 were both derivatized and radiolabeled according to this procedure and compared to the same antibodies derivatized and labeled using the six reaction steps comprising active ester strategy described before. *In vitro*, the antibodies revealed significant differences concerning stability, depending on the derivatization method and the buffer used for storage [47]. It could be shown that the active ester-derivatized ^89^Zr-conjugate had a much higher stability upon storage in buffers (0.25 M sodium acetate + 5 mg gentisic acid/ml buffer at pH 5.5 and 0.9% NaCl + 5 mg gentisic acid/ml at pH 5.0) with a ≤ 5% release of ^89^Zr from the complex within 6 days. In contrast to these findings, the isothiocyanate-modified antibodies ^89^Zr-DFO-SCN-mAb showed comparably low stabilities under the same conditions, liberating 10.5 ± 2.1% ^89^Zr upon storage in 0.25 M sodium acetate + 5 mg gentisic acid/ml buffer at pH 5.5 and even 48.4 ± 5.7% upon storage in 0.9% NaCl + 5 mg gentisic acid/ml at pH 5.0 after 6 days. It was suggested by the authors that this limited stability of the isothiocyanate-derivatized compounds could be a result of a radiation-induced formation of reactive hypochlorite ions affecting the integrity of the isothiocyanate [47]. Interestingly, the storage of the isothiocyanate-modified and ^89^Zr-labeled antibody in serum resulted in a release of only ~5% of ^89^Zr from the complex after 7 days. Confirming these *in vitro* results obtained in serum, the subsequently performed *in vivo* experiments in tumor-bearing mice revealed similar *in vivo* biodistribution properties together with a ~5% sternum accumulation at 72 h *p.i.* for both antibodies modified by isothiocyanate- and acid-amide-conjugation. As the isothiocyanate methodology does not seem to negatively influence the obtained *in vivo* results of ^89^Zr-labeled compounds although showing a significant instability upon storage in some buffers, several studies were performed recently using this simpler conjugation and radiolabeling approach [27,30,60,61,62,63].

## 4. Stability of the ^89^Zr-Desferrioxamine B Complex

In contrast to the ^89^Zr-desferrioxamine B complex, which is quickly cleared via the renal system when cleaved from the targeting biomolecule *in vivo* [37,39,46], ^89^Zr^4+^ liberated from the complex is first incorporated into plasma proteins [38] and later embedded into mineral bone [37,39,40] comparable to other large radiometal ions such as ^90^Y or ^177^Lu [40,64]. The accumulation of radiometal ions can result in bone marrow ablation [65,66] and thus should be reduced to an absolute minimum. DFO has not only been used for ^89^Zr-complexation in the past, but also for that of other radionuclides such as gallium and indium isotopes [67,68,69,70], but has been replaced in these cases by other chelators due to the observed instability of the formed complexes. In case of ^89^Zr, the stability of the complex is also limited as could be shown *in vitro* and *in vivo* in several studies, which is reflected in a bone accumulation ranging from 3 to 15% at 72–168 h *p.i.* [27,29,46,56,59,71].

This limited stability of the ^89^Zr-DFO complex can be explained by the structure of desferrioxamine B, being a linear and not a macrocyclic ligand. This is corroborated by a study comparing antibodies that were modified with either linear or macrocyclic chelators and radiolabeled with ^88^Y, ^177^Lu and ^89^Zr. As anticipated, the complexes with linear chelators (^88^Y-DTPA, ^177^Lu-DTPA and ^89^Zr-DFO) showed a comparably low stability of the complexes, whereas the macrocyclic ^88^Y-DOTA and ^177^Lu-DOTA complexes showed a high stability even after an incubation period of 16 days in human serum [72]. In other studies comparing ^88^Y- and ^177^Lu-DOTA to ^89^Zr-DFO-labeled antibodies, the lower stability of the ^89^Zr-DFO complex compared to the ^88^Y- and ^177^Lu-DOTA complexes was obvious as well, which was reflected in a significantly higher bone accumulation of ^89^Zr compared to ^88^Y and ^177^Lu [19,53,58]. Other experiments, in which the specific binding of the applied ^89^Zr-radiopharmaceuticals was prevented by blocking, showed an even more pronounced effect of the liberation of ^89^Zr from the complex and its accumulation in mineral bone [23,46,59,61].

Another factor influencing the complex stability is the correlation between the size of the radiometal ion and the cavity size that is formed by the chelator. If cavity size and metal ion radius are similar, stable complexes are formed that are normally characterized by a complete envelopment of the radiometal by the chelator, thus exhibiting no weak point in complex geometry that would allow for a complex challenge. Vice versa, an incomplete envelopment was shown to result in less stable complexes [73,74,75]. DFO however is reported to not completely envelope the Zr^4+^ ion in the complex by occupying only six of the eight coordinate positions [46] in the assumedly preferred octadentate complex geometry [42]. The resulting complex stability is therefore expected to be limited, which can be confirmed by the experimental results obtained *in vivo*. Thus, it was proposed recently that a more stable chelating agent exhibiting four bidentate hydroxamates could be better-suited for a stable ^89^Zr complexation *in vivo* than the three hydroxamates-containing DFO [76].

In addition to the observed instability of the ^89^Zr-DFO complex itself, the linker chemistry that is used for DFO-conjugation has been shown to have a subordinate but yet measurable effect on the stability of the ^89^Zr-DFO complex. It has been shown for example in an *in vivo* study comparing ^89^Zr-DFO-trastuzumab conjugates obtained using different DFO conjugation strategies, that the radioactivity accumulation in mineral bone depended on the linker chemistry used [55] and decreased in the following order: ^89^Zr-DFO-Chx-Mal mAb (conjugation via thiol-maleimide coupling, Scheme 3) > ^89^Zr-DFO-Ac-mAb (conjugation via bromoacetyl-thiol- and iodoacetyl-thiol-coupling, Scheme 3) > ^89^Zr-DFO-Bz-SCN-mAb (conjugation via isothiocyanate coupling, Scheme 6) > ^89^Zr-N-SucDFO-mAb (conjugation via acid-amide coupling, Scheme 5). This observed effect might be a result of the functional groups–such as thiol ethers or thio-ureas–compromising the integrity of the ^89^Zr-DFO complex by acting as additional ligands disturbing the ^89^Zr-DFO coordination sphere and resulting in an enhanced probability of complex-challenge by other potential ligands.

### 5. ^89^Zr-labeled Bioactive Compounds

As the favorable properties of ^89^Zr can only be fully exploited in imaging applications of compounds exhibiting slow *in vivo* pharmacokinetics, mostly ^89^Zr-labeled antibodies have been described so far. In case of smaller bioactive compounds with fast pharmacokinetics such as small molecules and peptides, other radionuclides such as ^18^F or ^68^Ga are commonly applied due to their more favorable physical decay characteristics such as a higher positron emission probability, γ-radiation purity and lower radiation doses due to the shorter half-lives of these nuclides. Nevertheless, not only antibodies labeled with ^89^Zr have been described and the following sections give an overview of the ^89^Zr-labeled bioactive compounds studied so far and their application in preclinical and/or clinical settings. Unless otherwise stated, the mentioned studies are preclinical.

#### 5.1. Peptides, Antibody Fragments and Serum Proteins

So far, only few examples of ^89^Zr-labeled peptides can be found. This is due to the generally fast pharmacokinetics of this substance class that enables a successful PET imaging using other more common nuclides such as ^18^F or ^68^Ga for radiolabeling. However, when evaluating peptide multimers, the use of ^89^Zr becomes more reasonable as the pharmacokinetics of these relatively large bioactive compounds can be rather slow [77,78]. Thus, one study evaluating DFO-Bz-SCN-derivatized and ^89^Zr-labeled c(RGDfK) monomers and dimers *in vitro* and *in vivo* was published recently. In this study, the anticipated results of higher *in vitro* binding avidity and *in vivo* tumor uptake together with higher tumor-to-background-ratios were found for the dimers compared to the respective monomers. As also observed in other studies using ^89^Zr-labeled biomolecules, a moderate bone accumulation was found in the *in vivo* evaluation that increased over the study period of 24h which can be attributed to liberated ^89^Zr [30]. Although the peptide dimers evaluated in these experiments could also have been labeled with other radiometal nuclides with shorter half-lives such as ^68^Ga or ^64^Cu, ^89^Zr could be a very favorable nuclide for evaluating larger peptidic multimers. 

Besides peptide multimers, the *in vivo* evaluation of antibody fragments could also profit from the long half-life of ^89^Zr enabling PET imaging at late time-points after tracer application. Thus, different nanobodies and an antibody fragment were labeled with ^89^Zr and successfully evaluated regarding their *in vivo* biodistribution and pharmacokinetics at late time-points [63,79]. Furthermore, ^89^Zr-labeling and subsequent PET imaging was also successfully used for an estimation of therapy efficiency and therapy planning in case of antibody fragments [80]. 

Apart from antibody fragments, also other proteins such as albumin and transferrin can be radiolabeled with ^89^Zr as was shown in a study using ^89^Zr-DFO-albumin for the successful nontargeted visualization of tumors through their increased vascularization and enhanced permeability and retention (EPR) effect [81]. In another study, the iron-transport protein transferrin was labeled with ^89^Zr to be applied in brain tumor imaging as well as in imaging of transferrin receptor expression [35]. While the ^89^Zr-transferrin showed favorable results in brain tumor imaging, exhibiting a higher tumor-to-brain ratio than ^18^F-fluorodeoxyglucose ([^18^F]FDG), it also showed high uptake in kidneys, liver and bone as well as inflammatory abscesses, hindering an efficient transferrin receptor detection in solid tumors [36].

#### 5.2. Nanoparticles and Microspheres

Besides peptides and medium scale proteins, ^89^Zr has also been used in radiolabeling and *in vivo* imaging of large synthetic structures such as microspheres, nanoparticles, nanotubes and liposomes. 

Examples include ^89^Zr-labeled microspheres (microspheres with a diameter of approximately 20–40 µm can be used in ^90^Y-labeled form in selective internal radiation therapy (SIRT) of tumors) that were evaluated regarding their *in vivo* biodistribution in terms of therapy planning and dosimetry purposes with PET before therapy with the ^90^Y-labeled particles takes place [32]. It was found in this study that of all the different diagnostic radiolabeled spheres (^64^Cu-, ^86^Y- and ^89^Zr-labeled microspheres were prepared and studied), the ^89^Zr-labeled derivatives were the most valuable PET imaging surrogates for the ^90^Y-labeled spheres and could be useful for therapy planning and dosimetry calculations due to their valuable *in vivo* PET imaging properties and the comparable half-lives of ^89^Zr and ^90^Y of 78.4 and 64.1 h, respectively. The ^86^Y-labeled spheres (t_1/2(^86^Y)_ = 14.7 h) were also shown to be useful for this application whereas the ^64^Cu-labelled derivatives showed an insufficient *in vitro* stability.

Nanocolloids of ^89^Zr-DFO-albumin were shown to be applicable for the detection of sentinel lymph nodes and were demonstrated to accumulate within several minutes in a rabbit lymphogenic metastasis model [82]. These nanocolloidal ^89^Zr-DFO-albumin particles where subsequently used in a continuing clinical study enabling a successful identification of tumorous sentinel lymph nodes even in direct vicinity to the primary tumor and yielding even more reliable results than the commonly applied ^99m^Tc-albumin nanocolloids [83]. Another example for ^89^Zr-labeled nanoparticles, short chain dextran nanoparticles (DNPs) that were intended for imaging of macrophages and inflammation, was recently described. Here, it could be shown that ^89^Zr-DFO-cross-linked dextran nanoparticles accumulated specifically in tissue resident macrophages within 24h of administration, which could also be useful for the detection of tumor associated macrophages (TAMs) in tumor diagnosis and therapy [31]. 

Further examples of ^89^Zr-labeled synthetic structures include single wall carbon nanotubes (SWCNT) which were shown to be useful as scaffold structures for surface-modification with targeting vectors and radionuclides due to their large surface and low immunogenicity. For example, carbon nanotubes derivatized with the tumor neovasculature-targeting antibody E4G10 and DFO were described. These nanotubes were subsequently labeled with ^89^Zr and evaluated using *in vivo* PET tumor imaging studies in tumor-bearing mice. The ^89^Zr-labeled constructs showed a rapid blood clearance and specific tumor accumulation but also a very high liver and kidney accumulation limiting the application of these constructs for imaging or therapy of tumors [33]. 

In addition to nanotubes, ^89^Zr-labeled liposomes were described recently. These liposomes, comprising the somatostatin receptor-affine peptide octreotide for tumor targeting, were labeled with ^89^Zr (for visualization with PET) as well as gadolinium ions (for visualization with MRI) to offer a platform for combined PET/MRI [34]. Although representing an interesting new approach for the design of multimodal and target-specific imaging agents, the *in vitro* stability of the liposomes was shown to be suboptimal: the incubation in mouse serum or in a solution of bovine serum albumin at 37 °C for 24 h to 96 h resulted in a release of approximately 50% of the radioactivity being a consequence of the direct labeling with ^89^Zr without using a chelator in the lipid monolayer of the liposome.

#### 5.3. Antibodies

The main application of ^89^Zr is still the labeling of antibodies for preclinical as well as clinical PET imaging studies [18,84,85]. This can be attributed to the slow pharmacokinetics and target accumulation of antibodies which necessitate a radionuclide exhibiting a long half-life such as ^89^Zr. 

An important point in antibody derivatization and radiolabeling is the immunoreactivity of the obtained conjugates as only a preserved immunoreactivity can result in a high bioactivity of the radioimmunoconjugates allowing for a successful *in vivo* target visualization. However, the immunoreactivity was determined in only about half of all studies. When determined, the immunoreactivity in the vast majority of cases was in a reasonable range between 70 and 96% [26,27,28,46,47,58,62,79]. Furthermore, the antibody derivatization was shown to not result in an aggregation of the biomolecules over time [52,54]. 

A summary of ^89^Zr-labeled antibodies and their application in preclinical or clinical imaging studies is given in Table 1. ^89^Zr-labeling and subsequent *in vivo* imaging was shown to provide valuable information about antibody biodistribution properties via PET, allowing the visualization of target tissues (usually a tumor entity) as well as receptor-expression also enabling to assess the efficiency of tumor therapeutics and pre-therapeutic dosimetry [24,25,29,46,50,51,57,60,61,62,86,87,88,89,90,91,92].

The efficient *in vivo* tumor visualization using ^89^Zr-labeled antibodies in clinical studies could be shown to depend on the specific activity of the radiolabeled antibody. It was demonstrated in a human imaging study with patients suffering from metastatic breast cancer that the ideal time-point for imaging as well as the optimal specific activity of ^89^Zr-trastuzumab depend on the pretreatment the patient had received. In patients who had already received trastuzumab for treatment purposes, the best imaging results were obtained using a mixture of ^89^Zr-trastuzumab and 10 mg of the unlabeled antibody (otherwise, target saturation is likely), whereas a mixture of ^89^Zr-trastuzumab and 50 mg of the unlabeled antibody had to be applied in trastuzumab-naive patients (otherwise, the antibody was cleared too fast, hampering an adequate visualization of the lesions) and the best tumor-to-background-contrasts could be achieved 4–5 days after ^89^Zr-trastuzumab application [93]. 

Furthermore, some studies are available that directly compare ^89^Zr- and ^111^In-labeled antibodies in order to evaluate the potential of the ^89^Zr-labeled compounds as surrogates for the ^111^In-labeled ones in order to make use of the advantages of PET imaging versus SPECT. Although the ^89^Zr-labeled antibodies showed a significant liberation of the radionuclide from the complex reflected in higher bone and liver radioactivity accumulation, they were found to give almost identical *in vivo* biodistribution results regarding tumor accumulation and uptake into other tissues compared to the respective ^111^In-labeled analogs in preclinical studies [26,52,59,94]. 

The use of ^89^Zr-labeled substances is not limited to diagnostic imaging alone. ^89^Zr-labeled radiopharmaceuticals were also shown to be useful as surrogates for the respective therapeutic ^90^Y- or ^177^Lu-labeled antibodies in order to perform dosimetry calculations and therapy planning in preclinical as well as clinical studies [19,53,72,95].

**Table 1:** ^89^Zr-labeled antibodies used for diagnostic imaging or therapy planning in preclinical and clinical studies.

Antibody used	Epitope	Target tissue	Preclinical	Clinical
323/A3	17.1 A	squamous-cell carcinoma and others	[54]	
5A10	“free” prostate-specific antigen (fPSA)	osseous prostate cancer lesions	[87]	
7E11	prostate-specific membrane antigen (PSMA)	prostate cancer	[51]	
bevacizumab	vascular endothelial growth factor (VEGF)	tumor angiogenic vessels	[24,93,86,92,97]	
cetuximab	epidermal growth factor receptor	various cancers	[29,47,72,97]	
CD45R	B220	B cells	[48]	
cG250	G250	renal cell carcinoma (RCC)	[94]	
cU36	CD44v6	head and neck squamous cell carcinoma (HNSCC) human studies	[8,19,47,58]	[16,28]
DN30	c-Met receptor	gastric cancer, Met/head and neck cancer	[57]	
E48	22 kDa antigen	squamous-cell carcinoma and others	[54]	
E4G10	vascular endothelial cadherin (VE-cad)	tumor angiogenic vessels	[33]	
fresolimumab	Transforming growth factor-β (TGF-β)	highly invasive or metastatic tumors as glioblastomas and human breast cancer	[52]	
hRS7	epithelial glycoprotein-1 (EGP-1)	epithelial carcinomas	[59]	
ibritumomab tiuxetan (Zevalin)	CD20	NHL (non-Hodgkin’s lymphoma) human studies	[53]	[53,95]
J591	prostate-specific membrane antigen (PSMA)	prostate cancer	[22,46]	
Onartuzumab	hepatocyte growth factor receptor (Met)	gastric carcinoma and glioblastoma	[89]	
panitumumab	(EGFR/HER1)	colorectal cancer, head and neck tumors	[61,90,98]	
PGN635	phosphatidylserine	apoptosis	[91]	
R1507	insulin-like growth factor receptor 1	breast cancer	[71]	
ranibizumab	VEGF	tumor induced angiogenesis	[88]	
rituximab	CD20	malign lymphoma cells	[47,62]	
trastuzumab	human epidermal growth factor receptor 2 (HER2)	breast cancer, ovarian, colorectal carcinoma human studies	[23,25,26,27,50,55,56,96]	[93,99,100]
TRC105	CD105	angiogenic endothelial cells	[60,101]	

Another possible application of ^89^Zr-labeled drugs is in combined multimodal imaging using PET/OI (OI = optical imaging). This has been shown to be a successful approach by the example of a ^89^Zr- and 800CW-NIR dye-labeled TRC105 antibody that was used for a pre-surgical tumor detection and intraoperative optical visualization of tumor material [101].

## 6. Conclusions and Outlook

^89^Zr shows very promising results in the *in vivo* PET imaging of slowly accumulating radiopharmaceuticals. However, some challenges remain such as the high energy photons emitted by this nuclide which can result in background noise of the images as well as the limited stability of the current chelator complexes. Without any doubt, work will be carried out to develop chelators and conjugation strategies that allow for a stable ^89^Zr-introduction into biomolecules, yielding compounds with optimized properties for the imaging of biological processes in clinical imaging applications. In addition, improvements in image reconstruction will overcome the obstacles of image background noise. These developments will thus enable to exploit the full potential of ^89^Zr.

The numerous successful examples of imaging studies with ^89^Zr-labeled bioactive molecules show the general applicability of this radiometal nuclide for long-term *in vivo* PET imaging. Furthermore, GMP-compliant ^89^Zr is already commercially available which allows using this most favorable nuclide in clinical routine. With regards to individual patient-tailored diagnostics and therapies, the development of new target-specific compounds—and among these presumably many antibodies or other large biomolecules—is still under way. Thus, ^89^Zr possesses a high potential for PET imaging applications in the future.

## References

[1] Zhang Y., Hong H., Cai W. (2011). PET tracers based on Zirconium-89. Curr. Radiopharm..

[2] Severin G.W., Engle J.W., Barnhart T.E., Nickles R.J. (2011). Zr-89 Radiochemistry for positron emission tomography. Med. Chem..

[3] Nayak T.K., Brechbiel M.W. (2009). Radioimmunoimaging with longer-lived positron-emitting radionuclides: Potentials and challenges. Bioconjug. Chem..

[4] Deri M.A., Zeglis B.M., Francesconi L.C., Lewis J.S. (2013). PET imaging with Zr-89: From radiochemistry to the clinic. Nucl. Med. Biol..

[5] Holland J.P., Sheh Y.C., Lewis J.S. (2009). Standardized methods for the production of high specific-activity zirconium-89. Nucl. Med. Biol..

[6] Baillie A.D., Ledingham K.W.D., Lynch J.G., Campbell M. (1979). Electron capture to positron emission ratios in the allowed decay of 89Zr. J. Phys. G Nucl. Phys..

[7] Lubberink M., Herzog H. (2011). Quantitative imaging of I-124 and Y-86 with PET. Eur. J. Nucl. Med. Mol. I.

[8] Verel I., Visser G.W., Boellaard R., Stigter-van Walsum M., Snow G., van Dongen G.A. (2003). 89Zr immuno-PET: Comprehensive procedures for the production of 89Zr-labeled monoclonal antibodies. J. Nucl. Med..

[9] Meijs W.E., Herscheid J.D.M., Haisma H.J., Wijbrandts R., Vanlangevelde F., Vanleuffen P.J., Mooy R., Pinedo H.M. (1994). Production of highly pure no-carrier added Zr-89 for the labeling of antibodies with a positron emitter. Appl. Radiat. Isotopes.

[10] Taghilo M., Kakavand T., Rajabifar S., Sarabadani P. (2012). Cyclotron production of 89Zr: A potent radionuclide for positron emission tomography. Int. J. Phys. Sci..

[11] Dejesus O.T., Nickles R.J. (1990). Production and Purification of Zr-89, a Potential Pet Antibody Label. Appl. Radiat. Isotopes.

[12] Kasbollah A., Eu P., Cowell S., Deb P. (2013). Review on Production of 89Zr in a Medical Cyclotron for PET Radiopharmaceuticals. J. Nucl. Med. Technol..

[13] Sadeghi M., Enferadi M., Bakhtiari M. (2012). Accelerator production of the positron emitter zirconium-89. Ann. Nucl. Energy.

[14] Lobo E.D., Hansen R.J., Balthasar J.P. (2004). Antibody pharmacokinetics and pharmacodynamics. J. Pharm. Sci..

[15] Imai K., Takaoka A. (2006). Comparing antibody and small-molecule therapies for cancer. Nat. Rev. Cancer.

[16] Borjesson P.K., Jauw Y.W., de Bree R., Roos J.C., Castelijns J.A., Leemans C.R., van Dongen G.A., Boellaard R. (2009). Radiation dosimetry of 89Zr-labeled chimeric monoclonal antibody U36 as used for immuno-PET in head and neck cancer patients. J. Nucl. Med..

[17] Brix G., Lechel U., Glatting G., Ziegler S.I., Munzing W., Muller S.P., Beyer T. (2005). Radiation exposure of patients undergoing whole-body dual-modality 18F-FDG PET/CT examinations. J. Nucl. Med..

[18] van Dongen G.A.M.S., Visser G.W.M., Hooge M.N.L.D., De Vries E.G., Perk L.R. (2007). Immuno-PET: A navigator in monoclonal antibody development and applications. Oncologist.

[19] Verel I., Visser G.W., Boellaard R., Boerman O.C., van Eerd J., Snow G.B., Lammertsma A.A., van Dongen G.A. (2003). Quantitative 89Zr immuno-PET for *in vivo* scouting of 90Y-labeled monoclonal antibodies in xenograft-bearing nude mice. J. Nucl. Med..

[20] Herzog H., Tellmann L., Qaim S.M., Spellerberg S., Schmid A., Coenen H.H. (2002). PET quantitation and imaging of the non-pure positronemitting iodine isotope I-124. Appl. Radiat. Isotopes.

[21] Thorek D.L.J., Robertson R., Bacchus W.A., Hahn J., Rothberg J., Beattie B.J., Grimm J. (2012). Cerenkov imaging-a new modality for molecular imaging. Am. J. Nucl. Med. Mol. Imaging.

[22] Ruggiero A., Holland J.P., Lewis J.S., Grimm J. (2010). Cerenkov Luminescence Imaging of Medical Isotopes. J. Nucl. Med..

[23] Holland J.P., Normand G., Ruggiero A., Lewis J.S., Grimm J. (2011). Intraoperative Imaging of Positron Emission Tomographic Radiotracers Using Cerenkov Luminescence Emissions. Mol. Imaging.

[24] Nagengast W.B., de Vries E.G., Hospers G.A., Mulder N.H., de Jong J.R., Hollema H., Brouwers A.H., van Dongen G.A., Perk L.R., Lub-de Hooge M.N. (2007). *In vivo* VEGF imaging with radiolabeled bevacizumab in a human ovarian tumor xenograft. J. Nucl. Med..

[25] Munnink T.H.O., de Korte M.A., Nagengast W.B., Timmer-Bosscha H., Schroder C.P., de Jong J.R., van Dongen G.A.M.S., Jensen M.R., Quadt C., Lub-de Hooge M.N. (2010). Zr-89-trastuzumab PET visualises HER2 downregulation by the HSP90 inhibitor NVP-AUY922 in a human tumour xenograft. Eur. J. Cancer.

[26] Dijkers E.C.F., Kosterink J.G.W., Rademaker A.P., Perk L.R., van Dongen G.A.M.S., Bart J., de Jong J.R., de Vries E.G.E., Lub-de Hooge M.N. (2009). Development and Characterization of Clinical-Grade Zr-89-Trastuzumab for HER2/neu ImmunoPET Imaging. J. Nucl. Med..

[27] Chang A.J., DeSilva R., Jain S., Lears K., Rogers B., Lapi S. (2012). 89Zr-radiolabeled trastuzumab imaging in orthotopic and metastatic breast tumors. Pharmaceuticals.

[28] Borjesson P.K., Jauw Y.W., Boellaard R., de Bree R., Comans E.F., Roos J.C., Castelijns J.A., Vosjan M.J., Kummer J.A., Leemans C.R. (2006). Performance of immuno-positron emission tomography with zirconium-89-labeled chimeric monoclonal antibody U36 in the detection of lymph node metastases in head and neck cancer patients. Clin. Cancer Res..

[29] Aerts H.J.W.L., Dubois L., Perk L., Vermaelen P., van Dongen G.A.M.S., Wouters B.G., Lambin P. (2009). Disparity Between *In Vivo* EGFR Expression and (89)Zr-Labeled Cetuximab Uptake Assessed with PET. J. Nucl. Med..

[30] Jacobson O., Zhu L., Niu G., Weiss I.D., Szajek L.P., Ma Y., Sun X., Yan Y., Kiesewetter D.O., Liu S. (2011). MicroPET imaging of integrin alphavbeta3 expressing tumors using 89Zr-RGD peptides. Mol. Imaging Biol..

[31] Keliher E.J., Yoo J., Nahrendorf M., Lewis J.S., Marinelli B., Newton A., Pittet M.J., Weissleder R. (2011). Zr-89-Labeled Dextran Nanoparticles Allow *in vivo* Macrophage Imaging. Bioconjug. Chem..

[32] Avila-Rodriguez M.A., Selwyn R.G., Hampel J.A., Thornadsen B.R., DeJesus O.T., Converse A.K., Nickles R.J. (2007). Positron-emitting resin microspheres as surrogates of Y-90 SIR-Spheres: A radiolabeling and stability study. Nucl. Med. Biol..

[33] Ruggiero A., Villa C.H., Holland J., Sprinkle S.R., May C., Lewis J.S., Scheinberg D.A., McDevitt M.R. (2010). Imaging and treating tumor vasculature with targeted radiolabeled carbon nanotubes. Int. J. Nanomed..

[34] Abou D.S., Thorek D.L., Ramos N.N., Pinkse M.W., Wolterbeek H.T., Carlin S.D., Beattie B.J., Lewis J.S. (2013). (89)Zr-Labeled Paramagnetic Octreotide-Liposomes for PET-MR Imaging of Cancer. Pharm. Res..

[35] Evans M.J., Holland J.P., Rice S.L., Doran M.G., Cheal S.M., Campos C., Carlin S.D., Mellinghoff I.K., Sawyers C.L., Lewis J.S. (2013). Imaging Tumor Burden in the Brain with Zr-89-Transferrin. J. Nucl. Med..

[36] Holland J.P., Evans M.J., Rice S.L., Wongvipat J., Sawyers C.L., Lewis J.S. (2012). Annotating MYC status with Zr-89-transferrin imaging. Nat. Med..

[37] Meijs W.E., Haisma H.J., VanderSchors R., Wijbrandts R., VandenOever K., Klok R.P., Pinedo H.M., Herscheid J.D.M. (1996). A facile method for the labeling of proteins with zirconium isotopes. Nucl. Med. Biol..

[38] Sotogaku N., Endo K., Hirunuma R., Enomoto S., Ambe S., Ambe F. (1999). Biochemical reactions of various trace elements with blood components and transport proteins. J. Radioanal. Nucl. Ch..

[39] Abou D.S., Ku T., Smith-Jones P.M. (2011). *In vivo* biodistribution and accumulation of Zr-89 in mice. Nucl. Med. Biol..

[40] Amano R., Enomoto S., Nobuta M., Sakamoto M., Tsujioka R., Ambe F. (1996). Bone uptake of vanadium in mice: Simultaneous tracing of V, Se, Sr, Y, Zr, Ru and Rh using a radioactive multitracer. J. Trace Elem. Med. Biol..

[41] Meijs W.E., Herscheid J.D.M., Haisma H.J., Pinedo H.M. (1992). Evaluation of Desferal as a Bifunctional Chelating Agent for Labeling Antibodies with Zr-89. Appl. Radiat. Isotopes.

[42] Wadas T.J., Wong E.H., Weisman G.R., Anderson C.J. (2010). Coordinating radiometals of copper, Gallium, Indium, Yttrium, and zirconium for PET and SPECT imaging of disease. Chem. Rev..

[43] Kiss T., Farkas E. (1998). Metal-binding ability of desferrioxamine B. J. Inclus. Phenom. Mol..

[44] Hernlem B.J., Vane L.M., Sayles G.D. (1996). Stability constants for complexes of the siderophore desferrioxamine B with selected heavy metal cations. Inorg. Chim. Acta.

[45] Baroncelli F., Grossi G. (1965). The complexing power of hydroxamic acids and its effect on the behaviour of organic extractants in the reprocessing of irradiated fuels—I the complexes between benzohydroxamic acid and zirconium, iron (III) and uranium (VI). J. Inorg. Nucl. Chem..

[46] Holland J.P., Divilov V., Bander N.H., Smith-Jones P.M., Larson S.M., Lewis J.S. (2010). Zr-89-DFO-J591 for ImmunoPET of Prostate-Specific Membrane Antigen Expression *In Vivo*. J. Nucl. Med..

[47] Perk L.R., Vosjan M.J.W.D., Visser G.W.M., Budde M., Jurek P., Kiefer G.E., van Dongen G.A.M.S. (2010). p-Isothiocyanatobenzyl-desferrioxamine: A new bifunctional chelate for facile radiolabeling of monoclonal antibodies with zirconium-89 for immuno-PET imaging. Eur. J. Nucl. Med. Mol. I.

[48] Walther M., Gebhardt P., Grosse-Gehling P., Wurbach L., Irmler I., Preusche S., Khalid M., Opfermann T., Kamradt T., Steinbach J. (2011). Implementation of Zr-89 production and in vivo imaging of B-cells in mice with Zr-89-labeled anti-B-cell antibodies by small animal PET/CT. Appl. Radiat. Isotopes.

[49] Vosjan M.J., Perk L.R., Visser G.W., Budde M., Jurek P., Kiefer G.E., van Dongen G.A. (2010). Conjugation and radiolabeling of monoclonal antibodies with zirconium-89 for PET imaging using the bifunctional chelate p-isothiocyanatobenzyl-desferrioxamine. Nat. Protoc..

[50] Holland J.P., Caldas-Lopes E., Divilov V., Longo V.A., Taldone T., Zatorska D., Chiosis G., Lewis J.S. (2010). Measuring the Pharmacodynamic Effects of a Novel Hsp90 Inhibitor on HER2/neu Expression in Mice Using Zr-89-DFO-Trastuzumab. PLoS One.

[51] Ruggiero A., Holland J.P., Hudolin T., Shenker L., Koulova A., Bander N.H., Lewis J.S., Grimm J. (2011). Targeting the Internal Epitope of Prostate-Specific Membrane Antigen with Zr-89-7E11 Immuno-PET. J. Nucl. Med..

[52] Munnink T.H.O., Arjaans M.E.A., Timmer-Bosscha H., Schroder C.P., Hesselink J.W., Vedelaar S.R., Walenkamp A.M.E., Reiss M., Gregory R.C., Lub-de Hooge M.N. (2011). PET with the Zr-89-Labeled Transforming Growth Factor-beta Antibody Fresolimumab in Tumor Models. J. Nucl. Med..

[53] Perk L.R., Visser O.J., Walsum M.S.V., Vosjan M.J.W.D., Visser G.W.M., Zijlstra J.M., Huijgens P.C., van Dongen G.A.M.S. (2006). Preparation and evaluation of Zr-89-Zevalin for monitoring of Y-90-Zevalin biodistribution with positron emission tomography. Eur. J. Nucl. Med. Mol. I.

[54] Meijs W.E., Haisma H.J., Klok R.P., vanGog F.B., Kievit E., Pinedo H.M., Herscheid J.D.M. (1997). Zirconium-labeled monoclonal antibodies and their distribution in tumor-bearing nude mice. J. Nucl. Med..

[55] Tinianow J.N., Gill H.S., Ogasawara A., Flores J.E., Vanderbilt A.N., Luis E., Vandlen R., Darwish M., Junutula J.R., Williams S.P. (2010). Site-specifically 89Zr-labeled monoclonal antibodies for ImmunoPET. Nucl. Med. Biol..

[56] Zeglis B.M., Mohindra P., Weissmann G.I., Divilov V., Hilderbrand S.A., Weissleder R., Lewis J.S. (2011). Modular strategy for the construction of radiometalated antibodies for positron emission tomography based on inverse electron demand Diels-Alder click chemistry. Bioconjug. Chem..

[57] Perk L.R., Walsum M.S.V., Visser G.W.M., Kloet R.W., Vosjan M.J.W.D., Leemans C.R., Giaccone G., Albano R., Comoglio P.M., van Dongen G.A.M.S. (2008). Quantitative PET imaging of Met-expressing human cancer xenografts with Zr-89-labelled monoclonal antibody DN30. Eur. J. Nucl. Med. Mol. I.

[58] Verel I., Visser G.W.M., Boerman O.C., van Eerd J.E.M., Finn R., Boellaard R., Vosjan M.J.W.D., Walsum M.S.V., Snow G.B., van Dongen G.A.M.S. (2003). Long-lived positron emitters zirconium-89 and iodine-124 for scouting of therapeutic radioimmunoconjugates with PET. Cancer Biother. Radiol..

[59] van Rij C.M., Sharkey R.M., Goldenberg D.M., Frielink C., Molkenboer J.D.M., Franssen G.M., van Weerden W.M., Oyen W.J.G., Boerman O.C. (2011). Imaging of prostate cancer with Immuno-PET and Immuno-SPECT using a radiolabeled anti-EGP-1 monoclonal antibody. J. Nucl. Med..

[60] Hong H., Severin G.W., Yang Y.A., Engle J.W., Zhang Y., Barnhart T.E., Liu G., Leigh B.R., Nickles R.J., Cai W.B. (2012). Positron emission tomography imaging of CD105 expression with Zr-89-Df-TRC105. Eur. J. Nucl. Med. Mol. I.

[61] Nayak T.K., Garmestani K., Milenic D.E., Brechbiel M.W. (2012). PET and MRI of metastatic peritoneal and pulmonary colorectal cancer in mice with human epidermal growth factor receptor 1-targeted Zr-89-labeled panitumumab. J. Nucl. Med..

[62] Natarajan A., Habte F., Gambhir S.S. (2012). Development of a novel long-lived immunopet tracer for monitoring lymphoma therapy in a humanized transgenic mouse model. Bioconjug. Chem..

[63] Vosjan M.J.W.D., Vercammen J., Kolkman J.A., Stigter-van Walsum M., Revets H., van Dongen G.A.M.S. (2012). Nanobodies targeting the hepatocyte growth factor: Potential new drugs for molecular cancer therapy. Mol. Cancer Ther..

[64] O'Mara R.E., McAfee J.G., Subramanian G. (1969). Rare earth nuclides as potential agents for skeletal imaging. J. Nucl. Med..

[65] Wright E.G. (1998). Radiation-induced genomic instability in haemopoietic cells. Int. J. Radiat. Biol..

[66] Banfi A., Bianchi G., Galotto M., Cancedda R., Quarto R. (2001). Bone marrow stromal damage after chemo/radiotherapy: Occurrence, Consequences and possibilities of treatment. Leuk. Lymph..

[67] Otsuka F.L., Fleischman J.B., Welch M.J. (1986). Comparative studies using 125I- and 111In-labeled monoclonal antibodies. Int. J. Radiat. Appl. Instr..

[68] Koizumi M., Endo K., Kunimatsu M., Sakahara H., Nakashima T., Kawamura Y., Watanabe Y., Saga T., Konishi J., Yamamuro T. (1988). Ga-67-labeled antibodies for immunoscintigraphy and evaluation of tumor targeting of drug-antibody conjugates in mice. Cancer Res..

[69] Smithjones P.M., Stolz B., Bruns C., Albert R., Reist H.W., Fridrich R., Macke H.R. (1994). Gallium-67/Gallium-68-[Dfo]-Octreotide—a Potential Radiopharmaceutical for Pet Imaging of Somatostatin Receptor-Positive Tumors-Synthesis and Radiolabeling *in-Vitro* and Preliminary *in-Vivo* Studies. J. Nucl. Med..

[70] OgiharaUmeda I., Sasaki T., Kojima S., Nishigori H. (1996). Optimal radiolabeled liposomes for tumor imaging. J. Nucl. Med..

[71] Heskamp S., van Laarhoven H.W.M., Molkenboer-Kuenen J.D.M., Franssen G.M., Versleijen-Jonkers Y.M.H., Oyen W.J.G., van der Graaf W.T.A., Boerman O.C. (2010). ImmunoSPECT and immunopet of IGF-1R expression with the radiolabeled antibody R1507 in a triple-negative breast cancer model. J. Nucl. Med..

[72] Perk L.R., Visser G.W.M., Vosjan M.J.W.D., Stigter-van Walsum M., Tijink B.M., Leemans C.R., van Dongen G.A.M.S. (2005). Zr-89 as a PET surrogate radioisotope for scouting biodistribution of the therapeutic radiometals Y-90 and Lu-117 in tumor-bearing nude mice after coupling to the internalizing antibody cetuximab. J. Nucl. Med..

[73] Woodin K.S., Heroux K.J., Boswell C.A., Wong E.H., Weisman G.R., Niu W.J., Tomellini S.A., Anderson C.J., Zakharov L.N., Rheingold A.L. (2005). Kinetic inertness and electrochemical behavior of copper(II) tetraazamacrocyclic complexes: Possible implications for *in vivo* stability. Eur. J. Inorg. Chem..

[74] Heroux K.J., Woodin K.S., Tranchemontagne D.J., Widger P.C.B., Southwick E., Wong E.H., Weisman G.R., Tomellini S.A., Wadas T.J., Anderson C.J. (2007). The long and short of it: The influence of N-carboxyethyl versus N-carboxymethyl pendant arms on *in vitro* and *in vivo* behavior of copper complexes of cross-bridged tetraamine macrocycles. Dalton Trans..

[75] Wangler B., Schirrmacher R., Bartenstein P., Wangler C. (2011). Chelating agents and their use in radiopharmaceutical sciences. Mini Rev. Med.Chem..

[76] Guerard F., Lee Y.S., Tripier R., Szajek L.P., Deschamps J.R., Brechbiel M.W. (2013). Investigation of Zr(IV) and Zr-89(IV) complexation with hydroxamates: Progress towards designing a better chelator than desferrioxamine B for immuno-PET imaging. Chem. Commun..

[77] Liu S. (2006). Radiolabeled multimeric cyclic RGD peptides as integrin alpha(v)beta(3) targeted radiotracers for tumor imaging. Mol. Pharm..

[78] Li Z.B., Cai W.B., Cao Q.Z., Chen K., Wu Z.H., He L.N., Chen X.Y. (2007). 64Cu-Labeled Tetrameric and octameric RGD peptides for small-animal PET of Tumor alpha(v)beta(3) integrin expression. J. Nucl. Med..

[79] Hoeben B.A.W., Kaanders J.H.A.M., Franssen G.M., Troost E.G.C., Rijken P.F.J.W., Oosterwijk E., van Dongen G.A.M.S., Oyen W.J.G., Boerman O.C., Bussink J. (2010). PET of hypoxia with Zr-89-labeled cG250-F(ab ')(2) in head and neck tumors. J. Nucl. Med..

[80] Munnink T.H.O., de Vries E.G.E., Vedelaar S.R., Timmer-Bosscha H., Schroder C.P., Brouwers A.H., Lub-de Hooge M.N. (2012). Lapatinib and 17AAG reduce Zr-89-trastuzumab-F(ab')(2) uptake in SKBR3 tumor xenografts. Mol. Pharm..

[81] Heneweer C., Holland J.P., Divilov V., Carlin S., Lewis J.S. (2011). Magnitude of enhanced permeability and retention effect in tumors with different phenotypes: Zr-89-albumin as a model system. J. Nucl. Med..

[82] Heuveling D.A., Visser G.W.M., Baclayon M., Roos W.H., Wuite G.J.L., Hoekstra O.S., Leemans C.R., de Bree R., van Dongen G.A.M.S. (2011). Zr-89-Nanocolloidal albumin-based PET/CT lymphoscintigraphy for sentinel node detection in head and neck cancer: preclinical results. J. Nucl. Med..

[83] Heuveling D.A., van Schie A., Vugts D.J., Hendrikse N.H., Yaqub M., Hoekstra O.S., Karagozoglu K.H., Leemans C.R., van Dongen G.A., de Bree R. (2013). Pilot study on the feasibility of PET/CT lymphoscintigraphy with 89Zr-nanocolloidal albumin for sentinel node identification in oral cancer patients. J. Nucl. Med..

[84] Vugts D.J., Visser G.W., van Dongen G.A. (2013). 89)Zr-PET radiochemistry in the development and application of therapeutic monoclonal antibodies and other biologicals. Curr. Top. Med. Chem..

[85] van Dongen G.A.M.S., Vosjan M.J.W.D. (2010). Immuno-positron emission tomography: Shedding light on clinical antibody therapy. Cancer Biother. Radiol..

[86] Nagengast W.B., de Korte M.A., Munnink T.H.O., Timmer-Bosscha H., den Dunnen W.F., Hollema H., de Jong J.R., Jensen M.R., Quadt C., Garcia-Echeverria C. (2010). (89)Zr-bevacizumab PET of early antiangiogenic tumor response to treatment with HSP90 inhibitor NVP-AUY922. J. Nucl. Med..

[87] Ulmert D., Evans M.J., Holland J.P., Rice S.L., Wongvipat J., Pettersson K., Abrahamsson P.A., Scardino P.T., Larson S.M., Lilja H. (2012). Imaging androgen receptor signaling with a radiotracer targeting free prostate-specific antigen. Cancer Discov..

[88] Nagengast W.B., Lub-de Hooge M.N., Oosting S.F., den Dunnen W.F., Warnders F.J., Brouwers A.H., de Jong J.R., Price P.M., Hollema H., Hospers G.A. (2011). VEGF-PET imaging is a noninvasive biomarker showing differential changes in the tumor during sunitinib treatment. Cancer Res..

[89] Jagoda E.M., Lang L.X., Bhadrasetty V., Histed S., Williams M., Kramer-Marek G., Mena E., Rosenblum L., Marik J., Tinianow J.N. (2012). Immuno-PET of the hepatocyte growth factor receptor Met using the 1-Armed antibody onartuzumab. J. Nucl. Med..

[90] Chang A.J., De Silva R.A., Lapi S.E. (2013). Development and characterization of 89Zr-labeled panitumumab for immuno-positron emission tomographic imaging of the epidermal growth factor receptor. Mol. Imaging.

[91] Ogasawara A., Tinianow J.N., Vanderbilt A.N., Gill H.S., Yee S., Flores J.E., Williams S.P., Ashkenazi A., Marik J. (2013). ImmunoPET imaging of phosphatidylserine in pro-apoptotic therapy treated tumor models. Nucl. Med. Biol..

[92] van der Bilt A.R., Terwisscha van Scheltinga A.G., Timmer-Bosscha H., Schroder C.P., Pot L., Kosterink J.G., van der Zee A.G., Lub-de Hooge M.N., de Jong S., de Vries E.G. (2012). Measurement of tumor VEGF-A levels with 89Zr-bevacizumab PET as an early biomarker for the antiangiogenic effect of everolimus treatment in an ovarian cancer xenograft model. Clin. Cancer Res..

[93] Dijkers E.C., Oude Munnink T.H., Kosterink J.G., Brouwers A.H., Jager P.L., de Jong J.R., van Dongen G.A., Schroder C.P., Lub-de Hooge M.N., de Vries E.G. (2010). Biodistribution of 89Zr-trastuzumab and PET imaging of HER2-positive lesions in patients with metastatic breast cancer. Clin. Pharm. Ther..

[94] Brouwers A., Verel I., Van Eerd J., Visser G., Steffens M., Oosterwijk E., Corstens F., Oyen W., Van Dongen G., Boerman O. (2004). PET radioimmunoscintigraphy of renal cell cancer using 89Zr-labeled cG250 monoclonal antibody in nude rats. Cancer Biother. Radiopharm..

[95] Rizvi S.N.F., Visser O.J., Vosjan M.J.W.D., van Lingen A., Hoekstra O.S., Zijlstra J.M., Huijgens P.C., van Dongen G.A.M.S., Lubberink M. (2012). Biodistribution, radiation dosimetry and scouting of Y-90-ibritumomab tiuxetan therapy in patients with relapsed B-cell non-Hodgkin's lymphoma using Zr-89-ibritumomab tiuxetan and PET. Eur. J. Nucl. Med. Mol. I.

[96] van Scheltinga A.G.T.T., van Dam G.M., Nagengast W.B., Ntziachristos V., Hollema H., Herek J.L., Schroder C.P., Kosterink J.G.W., Lub-de Hoog M.N., de Vries E.G.E. (2011). Intraoperative near-infrared fluorescence tumor imaging with vascular endothelial growth factor and human epidermal growth factor receptor 2 targeting antibodies. J. Nucl. Med..

[97] Cohen R., Stammes M.A., de Roos I.H., Stigter-van Walsum M., Visser G.W., van Dongen G.A. (2011). Inert coupling of IRDye800CW to monoclonal antibodies for clinical optical imaging of tumor targets. EJNMMI Res..

[98] Bhattacharyya S., Kurdziel K., Wei L., Riffle L., Kaur G., Hill G.C., Jacobs P.M., Tatum J.L., Doroshow J.H., Kalen J.D. (2013). Zirconium-89 labeled panitumumab: A potential immuno-PET probe for HER1-expressing carcinomas. Nucl. Med. Biol..

[99] Munnink T.H.O., Dijkers E.C., Netters S.J., Lub-de Hooge M.N., Brouwers A.H., Haasjes J.G., Schroder C.P., de Vries E.G. (2010). Trastuzumab pharmacokinetics influenced by extent human epidermal growth factor receptor 2-positive tumor load. J. Clin. Oncol..

[100] Gaykema S.B.M., Brouwers A.H., Hovenga S., Lub-de Hooge M.N., de Vries E.G.E., Schroder C.P. (2012). Zirconium-89-trastuzumab positron emission tomography as a tool to solve a clinical dilemma in a patient with breast cancer. J. Clin. Oncol..

[101] Hong H., Zhang Y., Severin G.W., Yang Y.N., Engle J.W., Niu G., Nickles R.J., Chen X.Y., Leigh B.R., Barnhart T.E. (2012). Multimodality Imaging of Breast Cancer Experimental Lung Metastasis with Bioluminescence and a Monoclonal Antibody Dual-Labeled with Zr-89 and IRDye 800CW. Mol. Pharm..

